# MiR-106b promotes cell proliferation via targeting RB in laryngeal carcinoma

**DOI:** 10.1186/1756-9966-30-73

**Published:** 2011-08-08

**Authors:** Kemin Cai, Yu Wang, Xueli Bao

**Affiliations:** 1 Department of Otorhinolaryngology Head and Neck Surgery, Taizhou People's Hospital, Taizhou 225300, P.R. China

**Keywords:** laryngeal carcinoma, miR-106b, RB, cell proliferation

## Abstract

MiR-106b is frequently up-regulated in various types of human cancer including laryngeal carcinoma. However the underlying mechanism of miR-106b involved in laryngeal carcinoma remains elusive. Here we showed that reduction of miR-106b induced cell cycle G0/G1 arrest by targeting tumor suppressor RB in human laryngeal carcinoma cells. Further, Introducing RB cDNA without 3'UTR abrogated miR-106b-induced cell proliferation. Finally, there was an inverse relationship between RB and miR-106b expression in laryngeal carcinoma tissues. To our knowledge, these data indicate for the first time that miR-106b directly regulate cell cycle by targeting RB in laryngeal carcinoma and that miR-106b could be potential therapeutic approaches for laryngeal carcinoma.

## Background

Laryngeal carcinoma is a common head and neck malignancy with high incidence as it accounts for approximately 2.4% of new malignancies worldwide every year [1,2]. Despite recent advances in cancer treatment, the prognosis for patients with laryngeal carcinoma especially at advanced stage remains poor. Therefore, it is essential to investigate the mechanism involved in the development and progression of laryngeal carcinoma.

MicroRNAs (miRNAs) are a new class of small, non-coding RNAs and regulate gene expression by binding to the 3'-untranslated regions (3'UTRs) of specific mRNAs. miRNAs could function as oncogenic miRNAs or tumor suppressor miRNAs, playing crucial roles in the development and progression of carcer [3,4]. Recent studies have indicated that frequent deregulation of miRNA in laryngeal carcinoma [5,6]. Let-7a was significantly downregulated both in human laryngeal squamous cancer tissues and Hep-2 cells, and functions as a potential tumor suppressor in human laryngeal cancer [5]. Hui et al investigated the significance of miRNA in patients with locally advanced head and neck squamous cell carcinoma and identified that thirty-eight miRNAs were significantly differentially expressed between malignant versus normal tissues [6]. Of note, upregulation of miR-106b, miR-423, miR-20a, and miR-16 as well as downregulation of miR-10a were newly observed.

In present work, we determined the function of miR-106b involved in laryngeal carcinoma. Reduction of miR-106b by antisense oligonucleotides inhibited cell proliferation and induced cell cycle G0/G1 arrest in laryngeal carcinoma cells. Moreover, RB was a direct target of miR-106b by luciferase reporter assay. Introduction of RB cDNA without 3'UTR abrogated miR-106b-induced cell proliferation. Finally, there was an inverse correlation of expression of miR-106b and RB in laryngeal carcinoma tissues.

## Materials and methods

### Clinical sample collection

Twenty laryngeal carcinoma tissues used in this study were obtained from Taizhou People's Hospital in China. Specimens were snap-frozen in liquid nitrogen, incuding 10 laryngeal carcinomas with stage I and II, and 10 laryngeal carcinomas with stage III and IV. The collection and use of the patient samples were reviewed and approved by Institutional Ethics Committees, and written informed consent from all patients was appropriately obtained.

### Cell culture and transfection

Hep-2 and TU212 cells were purchased from Chinese Academy of Sciences Cell Bank. Cells were maintained in DMEM medium supplemented with 10% fetal bovine serum. Cells were transfected using Lipofectamine 2000 (Invitrogen, USA) at the time of 50-60% confluent. 48 h after transfection, cells were harvested for further studies.

### Plasmids and oligonucleotides

For expression plasmid construct, wild-type RB cDNA sequence without 3'UTR was selected and cloned into Pgenesil-1 vector. 2'-O-methyl (OMe)-oligonucleotides were chemically synthesized and purified by GenePharma Co., Ltd. (Shanghai, China). The amount of oligonucleotides transfected was 50 nmol/L. Sequences as follows: miR-106b, 5'- UAAAGUGCUGACAGUGCAGAU-3'; anti-miR-106b (As-miR-106b), 5'-AUCUGCACUGUCAGCACUUUA-3'; scrambled miRNA (negative control), 5'-UUGUACUACACAAAAGUACUG-3'.

### Real time PCR

Trizol reagent was used to isolate total RNA from cells 48 h after transfection. The RT-real-time PCR was carried out with the miRNA detection kit (Ambion, USA). Amplification reaction protocol was performed for 40 cycles consisting 95°C for 3 min, 95°C for 15 sec, 60°C for 30 sec. Both RT and PCR primer were purchased from Ambion. 5S RNA was used for normalization. Relative quantification was conducted using amplification efficiencies derived from cDNA standard curves and obtained relative gene expression. Relative gene expression was calculated via a 2^ΔΔCt ^method.

### MTT assay

Cells were plated at 10^4 ^cells per well in 96-well plates with six replicate wells. After transfection as described previously, 20 μl of MTT (5 g/L, Sigma, USA) was added into each well at each day of consecutive 4 days after treatment and the cells were incubated for additional 4 h, the supernatant was then discarded. 200 μl of DMSO was added to each well to dissolve the precipitate. Optical density (OD) was measured at wave length of 550 nm. The data are presented as the mean ± SD, which are derived from triplicate samples of at least three independent experiments.

### Cell cycle analysis

Cells were washed with PBS, fixed with 70% ethanol for at least 1 h. After extensive washing, the cells were suspended in HBSS (Hank's Balanced Salt Solution) containing 50 μg/mL PI and 50 μg/ml RNase A and incubated for 1 h at room temperature, and analyzed by FACScan (Becton Dickinson, USA). Cell cycle analysis was analyzed by ModFit software. Experiments were performed in triplicate. Results were presented as % of cell in a particular phase.

### Western blot analysis

Equal amounts of protein per lane were separated by 8% SDS-polyacrylamide gel and transferred to PVDF membrane. The membrane was blocked in 5% skim milk for 1 h and then incubated with a specific antibody for 2 h. The antibodies used in this study were: antibodies to RB (Santa Cruz, USA). The antibody against β-actin (Santa Cruz, USA) was used as control. The specific protein was detected by using a SuperSignal protein detection kit (Pierce, USA). The band density of specific proteins was quantified after normalization with the density of β-actin.

### Luciferase reporter assay

The human RB 3'UTR (bases 813-959) were amplified and cloned into the XbaI site of the pGL3-control vector (Promega, USA), downstream of the luciferase gene, to generate the plasmids pGL3-WT-RB-3'UTR. pGL3-MUT-RB-3'UTR plasmids were generated from pGL3-WT-RB-3'UTR by deleting the binding site (bases 883-889) for miR-106b "GCACUUU". For the luciferase reporter assay, cells were cultured in 96-well plates, transfected with the plasmids and As-miR-106b using Lipofectamine 2000. 48 h after transfection, luciferase activity was measured using the Dual Luciferase Reporter Assay System (Promega). Firefly luciferase activity was normalized to renilla luciferase activity for each transfected well.

### Statistical analysis

Statistics was determined by ANOVA, or t test using SPSS11.0. Statistical significance is determined as P < 0.05.

## Results

### MiR-106b expression in laryngeal carcinomas

**Figure 1:**
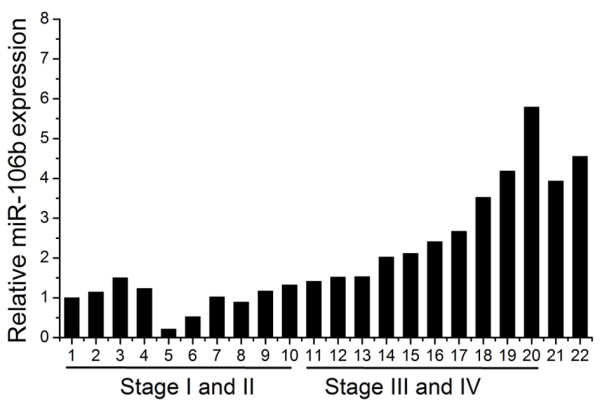
**Expression of miR-106b in laryngeal carcinoma**. Expression levels of miR-106b in laryngeal carcinoma tissues and cell lines (21: Hep-2 cells, 22: TU212 cells) were measured by Real time PCR and quantified as described in methods.

To explore miR-106b expression in laryngeal carcinomas, we examined 20 human laryngeal carcinoma specimens with different clinical stages using Real time PCR. As shown in Figure 1, the levels of miR-106b increased markedly in laryngeal carcinomas with stage III and IV in comparison to those with stage I and II (P < 0.01). And we also found high miR-106b expression in Hep-2 and TU212 laryngeal carcinoma cells (Figure 1).

### MiR-106b inhibition suppresses cell proliferation and induces G0/G1 arrest

**Figure 2:**
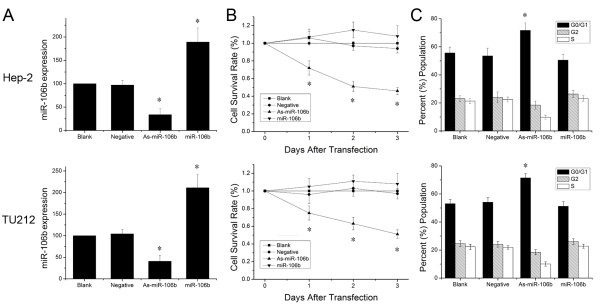
**Reduction of miR-106b suppressed laryngeal carcinoma cell proliferation**. (A) Expression levels of miR-106b in laryngeal carcinoma cells 48 h after As-miR-106b and miR-106b treatment. (B) MTT assay displayed that cells treated with As-miR-106b proliferated at a significantly lower rate than control groups after transfection. (C) After 48 h treatment, cells were harvested and performed by cell cycle assay. Data are expressed as the mean ± SD of 3 independent experiments. * P < 0.05 compared with control group.

As-miR-106b and miR-106b mimic oligonucleotides were employed to change miR-106b expression in Hep-2 and TU212 cells to evaluate the significance of miR-106b in laryngeal carcinoma. In both two cells, miR-106b expression significantly decreased in As-miR-106b group and increased in miR-106b group 48 h after transfection (Figure 2A). MTT assay data showed that a statistically significant cell proliferation inhibition was found in As-miR-106b group of Hep-2 cells, compared with control groups respectively. Similar trend was observed in TU212 cells (Figure 2B). There was no difference between blank control group and negative control group in the whole experiment. Next we analyzed the cell cycle distribution by FACS. As-miR-106b treated cells represented significant ascends in G0/G1 phase in comparison to untreated Hep-2 and TU212 cells (Figure 2C). However, we did not observe a significant difference in the rate of growth inhibition between miR-106b group and blank control group; although a slightly increasing trend of cell survival rate and G0/G1 phase was seen in Hep-2 and TU212 cells. These results raise the possibility that there exists a threshold value for miR-106b up-regulation. Taken together, reduction of miR-106b can induce cells arrest at G0/G1 phases, thereby inhibiting cell proliferation in laryngeal carcinoma cells.

### RB is a direct target of miR-106b

**Figure 3:**
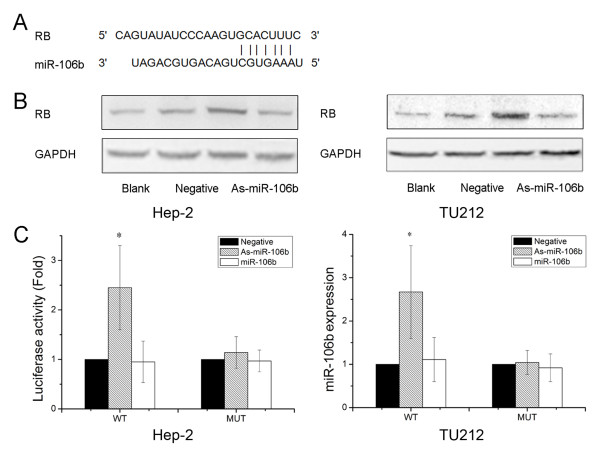
**RB was identified as target genes of miR-106b**. (A) A schematic representation showing the putative target site for miR-106b in RB mRNA 3'UTR. (B) Cells were transfected with As-miR-106b and miR-106b, and the expression of RB was analyzed by Western blot. The expression of β-actin was used as a loading control. (C) Luciferase constructs were transfected into cells transduced with As-miR-106b and miR-106b. Luciferase activity was determined 48 h after transfection. The ratio of normalized sensor to control luciferase activity is shown. Data are expressed as the mean ± SD of 3 independent experiments. * P < 0.05 compared with control group.

To further explore the molecular mechanism of As-miR-106b induced cell cycle in laryngeal carcinoma cells, bioinformatics analysis of miR-106b potential target genes was performed through the databases TargetScan http://www.targetscan.org and PicTar http://www.pictar.bio.nyu.edu, We found that tumor suppressor RB associated with cell cycle contained the highly conserved putative miR-106b binding sites (Figure 3A). To determine whether RB is directly regulated by miR-106b, Western blot analysis and Luciferase reporter assay were employed. Western blot analysis showed that a notable induction of RB expression was detected after knockdown of miR-106b in Hep-2 and TU212 cells (Figure 3B). Further, we created pGL3-WT-RB-3'UTR, and pGL3-MUT-RB-3'UTR plasmids. Reporter assay revealed that inhibition of miR-106b triggered a marked increase of luciferase activity of pGL3-WT-RB-3'UTR plasmid both in Hep-2 and TU212 cells, without change in luciferase activity of pGL3-MUT-RB-3'UTR (Figure 3C). These data indicate that RB is a direct target of miR-106b in laryngeal carcinoma.

### Core role of RB in miR-106b-mediated cell proliferation

**Figure 4:**
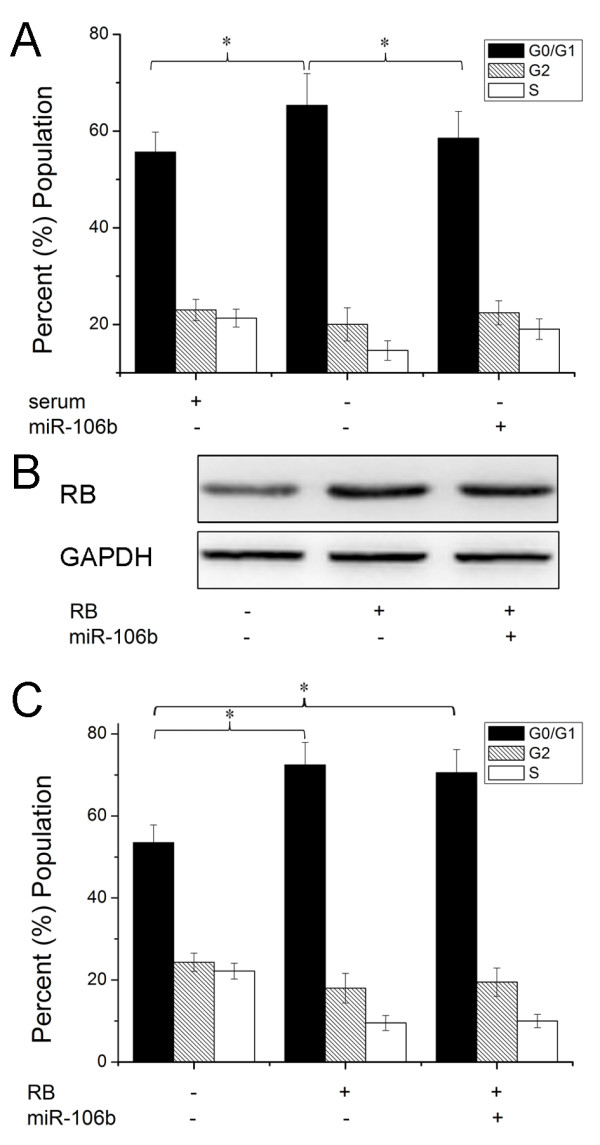
**Expression of RB abrogates miR-106b -induced cell proliferation**. (A) Cells were transfected with miR-106b and then treated with serum starvation and cell proliferation was performed by cell cycle analysis. (B) Cells were transfected with pcDNA-RB (without the 3'UTR) and miR-106b, RB protein level was detected by Western blot assay. β-actin protein was regarded as endogenous normalizer. (C) Cells were transfected with pcDNA-RB (without the 3'UTR) and miR-106b, cell cycle assay was performed respectively. Data are expressed as the mean ± SD of 3 independent experiments. * P < 0.05.

Having demonstrated RB as a direct target of miR-106b, we next examined the importance of RB in miR-106b-mediated cell proliferation. The cell cycle distribution analysis showed that upregulation of miR-106b significantly reduced cell cycle G0/G1 phase arrest induced by serum starvation (Figure 4A). Then we transfected Rb without 3'UTR into Hep-2 cells. Western blot assay showed that transfection with RB without 3'UTR overrided RB expression targeted by miR-106b (Figure 4B). As shown in Figure 4C, the cells transfected RB significantly induced G0/G1 phase arrest. However, when we transfected with RB without 3'UTR and miR-106b, expression of RB largely abrogated the effect of miR-106b on cell cycle distribution. These findings suggest that RB is a major target of miR-106b involved in laryngeal carcinoma cell proliferation.

### Inverse correlation of expression of miR-106b and RB in laryngeal carcinoma tissues

**Figure 5:**
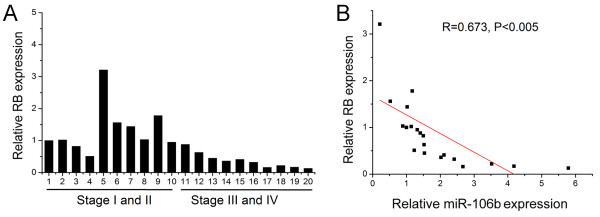
**MiR-106b inversely correlates with RB expression in laryngeal carcinoma tissues**. (A) Expression levels of RB in laryngeal carcinoma tissues were measured by Real time PCR and quantified as described in methods. (B) Inverse correlation of miR-106b expression with RB expression in laryngeal carcinoma tissues by Pearson correlation analysis. Data are presented as the means of triplicate experiments.

We further explored the correlation of between miR-106b and RB expression in laryngeal carcinomas. We tested RB expression in these 20 human laryngeal carcinoma specimens and found RB expression was down-regulated in laryngeal carcinomas with stage III and IV in comparison to those with stage I and II (Figure 5A). Further, Pearson correlation showed that a significant negative correlation existed between miR-106b and RB expression in laryngeal carcinoma tissues (R = 0.673, P < 0.005) (Figure 5B).

## Discussion

Recent evidences indicate that miR-106b has participated in development and progression of human tumors, such as hepatocellular cancer, prostate cancer, gastric cancers and renal cell carcinoma [7-10]. In this study, repression of miR-106b resulted in cell proliferation inhibition and cell cycle G0/G1 arrest in laryngeal carcinoma cells. Further, As-miR-106b regulated RB expression via targeting 3'UTR of RB. Finally, expression of RB abolished cell proliferation of miR-106b.

MiR-106b, located at Chr 7, is one member of miR-106b-25 cluster. Several genes have been evidenced to be the targets of miR-106b, such as p21/CDKN1A and TGF-β type II receptor (TβR II). Ivanovska et al reported that miR-106b gain of function promotes cell cycle progression, whereas loss of function reverses this phenotype. And p21/CDKN1A is a direct target of miR-106b and that its silencing plays a key role in miR-106b-induced cell cycle phenotypes [11]. In the pathogenesis of Alzheimer's diseases, miR-106b regulated TβR II expression via binding 3' UTR of the TβR II mRNA, thereby leads to impairment in TGF-β signaling [12]. Here, we evidenced that RB was a novel direct and functional target of miR-106b involved in cell proliferation of laryngeal carcinoma cells. Reduction of miR-106b regulated RB expression via targeting 3'UTR of RB, and expression of RB largely abrogated miR-106b-induced cell proliferation in laryngeal carcinoma cells. And miR-106b increased with the increasing stages of laryngeal carcinoma tissues, and inversely correlated with RB expression.

The RB-pathway, consisting of inhibitors and activators of cyclin-dependent kinases, the retinoblastoma tumor suppressor (RB), the E2F-family of transcription factors and cyclin-dependent protein kinases, plays critical roles in the regulation of cell cycle progression and cell death [13,14]. Components of this pathway, particularly RB, p16Ink4a, and cyclin D1, are frequently altered in human cancers to promote deregulated cellular proliferation [15,16]. Recently, a comprehensive analysis of the genome and transcriptome has shown that the RB-pathway is altered in 78% of the primary glioblastoma tumor samples [17]. In our study, RB was lower expression in laryngeal carcinomas with stage III and IV in comparison to those with stage I and II, in line with the previous study [18]. And upregulation of RB controls G1/S transition in the cell cycle. Up to now, the approaches that specifically target the RB-pathway have been used in preclinical models, but not yet in the clinical setting [19,20]. However, the RB-pathway is still a promising target in cancer intervention and further investigations are needed.

In conclusion, we have showed that miR-106b is one of oncogenic miRNAs in laryngeal carcinomas and RB is a novel and critical target of miR-106b. These results suggest that miR-106b might be useful as a potential therapeutic target for laryngeal carcinoma and more in depth analysis is required.

## Competing interests

The authors declare that they have no competing interests.

## Authors' contributions

CK have made substantial contributions to acquisition of data. WY participated in the design of the study and performed the statistical analysis. BX participated in its design and drafted the manuscript. All authors read and approved the final manuscript.
